# Developing a hierarchical priority framework for therapeutic landscapes in age-friendly community parks: a systematic review

**DOI:** 10.3389/fpubh.2026.1839266

**Published:** 2026-07-01

**Authors:** Xinyu Lin, Wardah Fatimah Mohammad Yusoff, Mohd Khairul Azhar Mat Sulaiman

**Affiliations:** 1 Department of Architecture and Built Environment, Faculty of Engineering and Built Environment, Universiti Kebangsaan Malaysia (The National University of Malaysia), Bangi, Selangor, Malaysia; 2 Faculty of Arts and Crafts, Meizhouwan Vocational Technology College, Putian, Fujian, China

**Keywords:** age-friendly design, community parks, hierarchical framework, older adults, therapeutic landscape

## Abstract

As aging-in-place policies gain global adoption, community parks are vital for older adults’ health. Therapeutic landscapes offer a promising framework for understanding how park environments support well-being. However, how these dimensions contribute to therapeutic outcomes and whether they operate through a structured hierarchy remain theoretically underdeveloped. The aim of this systematic review is to determine how therapeutic landscape dimensions collectively and sequentially support geriatric health. Following PRISMA guidelines, a systematic search of Web of Science and Scopus synthesized data from 25 studies across 14 countries. Two principal findings emerge. First, the interaction between older adults and park environments emerges as a cyclical process where users actively shape their therapeutic experiences. Second, a Hierarchical Priority Framework is proposed in which physical, social, activity and immediate health features form a foundation leading to the symbolic dimension. This symbolic level represents the highest therapeutic experience, reachable only through sustained engagement. The main conclusion establishes that physical infrastructure alone cannot sustain therapeutic value. Park design and policy must continuously support both physical spaces and social activities to maintain long-term health benefits.

## 1 Introduction

Population aging has emerged as one of the most significant demographic transitions of the twenty-first century. The number of people aged 60 and over is projected to reach 2.1 billion by 2050 (1), with East Asian countries, including China, experiencing the most rapid pace of aging globally. As aging-in-place policies gain widespread adoption, community parks have increasingly been recognized as critical infrastructure for supporting older adults’ health and well-being in everyday residential settings.

The theoretical framework of therapeutic landscapes, first articulated by Gesler (2), conceptualizes how physical, social, and symbolic dimensions of place contribute to healing experiences. This framework has been widely applied in environmental gerontology and public health research, generating substantial evidence on the health benefits of parks and natural environments for older adults.

Despite these contributions, three research gaps remain. First, the therapeutic landscape dimensions identified in existing literature have largely been treated as parallel constituents of therapeutic experience, rather than as elements operating in a structured sequence. Whether these dimensions function through a hierarchy in which some dimensions enable and condition others remain theoretically underdeveloped. Second, the relationship between therapeutic landscapes and older adults’ health has predominantly been framed as unidirectional, with environmental features producing health outcomes as endpoints. The role of older adults as active agents who reshape the conditions of their own therapeutic experience has been underexplored. Third, much of the existing therapeutic landscape theory has been developed in Western contexts, with limited attention to how cultural specificity, particularly in non-Western settings, shapes the relative importance of different therapeutic dimensions.

To address these gaps, this systematic review synthesizes evidence on therapeutic landscapes in community parks for older adults. The review pursues three research objectives:

(1) To examine whether the therapeutic landscape dimensions identified in community parks operate through a structured hierarchy, and to develop a framework that articulates their relative priority and interdependence.
(2) To reconceptualize the relationship between therapeutic landscapes and older adults’ health by examining how older adults function as active agents who reshape their own therapeutic environments through sustained engagement.
(3) To examine how cultural specificity, particularly in Asian contexts underrepresented in earlier therapeutic landscape research, shapes the relative importance of therapeutic landscape dimensions, and to draw implications for design and policy practice.

## 2 Literature review

### 2.1 Community/home-based eldercare is a mainstream phenomenon with increasing outdoor health requirements

The majority of older adults prefer community-based care, home-based care. In European Union countries, 90–95% of older adults aged 65 and over choose aging in place. In the United States, 77 percent of older adults express a preference for aging in place. In China, 97 percent of older participate in community-based care (3–5). The main reason is that older adults are accustomed to their familiar living space, community, friends, family and surroundings. The familiarity with the living environment provides them with a sense of belonging, security, and comfort, reducing the challenges during the aging process.

Research by the World Health Organization (WHO) has found that there are four factors influencing individual health and longevity: personal behavior and lifestyle factors, environmental factors, genetic factors, and healthcare service factors. Among these, personal behavior and lifestyle account for 60% of the overall impact. Fostering and preserving the physical health and functionality of the older adults can lead to a reduction in national fiscal expenditures on healthcare and long-term care, contributing to the maintenance of sustainable economic development (6). In order to achieve the goal of healthy aging and enable older adults to enter late life comfortably while maintaining good physical functioning, individuals need to maintain a healthy lifestyle, while governments and societies need to create a supportive environment.

Therefore, creating environments that promote individual healthy behaviors and lifestyles for older adults, along with providing beneficial social support and healthcare services, allows older adults to confidently engage and enjoy in various outdoor activities, maintain their health, and establish a positive cycle of well-being.

### 2.2 Community parks address outdoor health requirements of the older adults

Community parks play a significant role as the nearest outdoor activities and socializing places for older adults who prefer community-based care, home-based care in residential areas. This care model emphasizes the therapeutic benefits of community parks, which promote health and enhance quality of life.

As early as 2007, WHO emphasized Global Age-friendly Cities: A Guide the significant impact of urban green spaces and outdoor environments on older adults’ autonomy, improving their quality of life, and promoting social participation (7). The guide indicated that the friendliness of outdoor environments for older adults is evaluated based on several aspects, including pleasant and clean surroundings, good security, extensive greenery, readily available outdoor resting areas, sufficient restroom facilities, accessible pathways, and safe non-motorized lanes (sidewalks, bicycle lanes, pedestrian crossings), among others. Providing a supportive environment for older adults can eliminate their fear of hazardous environments, reduce outdoor falls, and encourage them to maintain their ability and confidence in engaging in outdoor activities (8, 9).

Placing greater emphasis on the importance of community parks directly advances the United Nations’ Sustainable Development Goals (SDGs). Specifically, SDG 11 (Sustainable Cities and Communities) calls for inclusive green public spaces, which in turn helps reduce inequalities as targeted by SDG 10. Ultimately, this fosters an environment that supports healthy aging and well-being, in line with SDG 3.

Compared with other types of parks, the greatest advantage is that community parks serve the closest outdoor natural public spaces that older adults can reach. It means that older adults are able to obtain equitable health opportunities. This also embodies fairness, a core indicator of urban care for older adults (SDG 10). Furthermore, individuals residing near green spaces experience significantly fewer mental distresses compared to those living farther away (10). Nutsford et al. (11) found that for every 100-meter reduction in the distance to the nearest urban green space, the risk of mental health problems decreases by 3% (11). People who live in areas with high accessibility and are closer to green regions experience less psychological distress (12, 13).

Community parks can play a key role in preventive health strategies by providing therapeutic characteristics (such as rehabilitation activities and physical exercises), consistent with the therapeutic features needed by older adults in home care environments (SDG 3). Older adults typically faces risks of chronic diseases such as pneumonia and heart disease, which can be mitigated through effective health interventions (14, 15). Community parks can serve as ideal extension sites for hospitals and home care settings for older adults. The natural environment of these parks complements indoor rehabilitation, allowing older adults to perform prescribed exercises and therapeutic activities in relaxing and accessible settings. This extension of rehabilitation from medical institutions to community parks supports continuity of care and may enhance rehabilitation outcomes through exposure to natural environments.

Community parks not only enable older adults to actively integrate into social life but also eliminate barriers for their participation in family life, community development, and public engagement. It also promotes intergenerational sharing as well as cultivating harmonious values, encouraging older adults and younger individuals to collaborate in building an age-friendly society. Community parks, as the public green space most closely connected to older adults, have the most obvious distance advantages.

### 2.3 Therapeutic landscape as a mechanism in community parks

Therapeutic landscapes are predominantly utilized in specialized settings such as hospitals and care homes. While some researchers have attempted to incorporate this concept into public spaces, such instances are not abundant. The intervention of therapeutic landscapes in public spaces proves beneficial for the community parks in the context of an aging society, offering a micro-level perspective with detailed design plans and assessment criteria (16). The micro-level focus is particularly crucial in addressing the diverse health needs associated with an aging population, aiming to create urban public green spaces that promote their well-being.

Community parks provide supportive spaces for older adults, while therapeutic landscapes serve as effective means to promote their health. Based on Gesler’s definition of therapeutic landscapes: “where the physical and built environments, social conditions and human perceptions combine to produce an atmosphere which is conducive to healing” (17). Therapeutic landscapes, centered around nature, focus on the relationship between health and the environment. By providing various dynamic stimuli to human sensory organs, therapeutic landscapes play a role in maintaining and promoting health (18). Thus, therapeutic landscapes represent a complex relationship between individuals and their broader social and environmental settings (19).

### 2.4 Dimensions of therapeutic landscape

The therapeutic quality of landscapes is assessed based on three dimensions: physical, social, and symbolic (2). Smith et al. (20) evaluated whether the Glasgow Canal and its surrounding landscape possessed therapeutic landscape qualities using a framework developed by Völker and Kistemann (21), which incorporated an additional activity dimension. This addition was justified by the strong association between activity and health. Community parks, as significant locations for recreational activities, play a crucial role in the health and well-being of older adults. Therefore, the evaluation of whether a community park possesses therapeutic landscape qualities can be conducted based on the dimensions of physical environment, social environment, activity environment and symbolic.

The physical dimension encompasses natural elements and built features, including accessible pathways, seating, and exercise facilities, that reduce stress, enhance mood, and support both passive restoration and active rehabilitation (22–24). The social dimension operates through informal encounters, group activities, and intergenerational interactions that reduce isolation, build community, and promote active aging (25, 26). The activity dimension refers to programmed and spontaneous engagement such as organized exercise, leisure, and recreational pursuits that create opportunities for physical, social, and mental health promotion across different ability levels (27, 28). The symbolic dimension encompasses the cultural meanings, memories, and emotional attachments older adults associate with a place, shaping how they perceive and invest in therapeutic spaces and fostering place attachment and spiritual comfort (17, 18).

Through the integration of physical, social, activity, and symbolic environments, therapeutic landscapes in community parks can create a comprehensive healing environment that supports older adults’ physical health, social connections, meaningful engagement, and emotional well-being to enhance age-friendly urban city environments.

## 3 Methods

### 3.1 Search strategy

This systematic review followed PRISMA guidelines, searching Web of Science and Scopus for English-language journal articles published before 31 July 2025. The search included English journal articles. Screening was conducted manually against the eligibility criteria outlined in Table 1, with no restrictions on country or region of study. The full search strings, including all keywords, Boolean operators, truncations and field tags, are detailed in Supplementary material S1.

**Table 1:** Inclusion and exclusion criteria.

Inclusion criteria	Exclusion criteria
(1) the study fell within the field of health geography, urban studies, or a related discipline; (2) the study reported outcomes related to the health of older adults; (3) the study site was a community park situated in a residential area; (4) the study addressed research theory, phenomena, or design practice relevant to therapeutic landscapes for older adults; (5) the study explicitly linked landscape qualities or characteristics to at least one dimension of older adults’ health; (6) the study employed qualitative, quantitative, or mixed methods, with a clearly reported sample size and study location; (7) the article was published as a peer reviewed journal article in English	(1) were duplicate records identified across databases; (2) were published as encyclopedia entries, book chapters, book reviews, conference abstracts, correspondence, data articles, discussions, editorials, mini reviews, or short communications; (3) were published in a language other than English; (4) fell outside the field of health geography or urban studies; (5) reported outcomes unrelated to the health of older adults; (6) did not address research theory, phenomena, or design practice relevant to the review focus; (7) focused on clinical or institutionalized older adult subgroups (e.g., hospitalized patients, bedridden individuals, or those with late stage dementia), rather than community dwelling older adults; (8) were earlier versions of studies drawing on the same sample and dataset, in which case only the most recently published version was retained; (9) did not explicitly link landscape qualities or characteristics to health dimensions; (10) did not employ qualitative, quantitative, or mixed methods; (11) did not specify sample size or study site

Conceptual relations among the space-related search terms were carefully defined to ensure both comprehensiveness and precision. In this study, “community park,” “urban park,” “public park,” and “neighborhood park” are treated as functionally equivalent under the overarching concept of urban public green spaces. Although urban planning hierarchy distinctions exist regarding their spatial scales and service radii, these variations are theoretically collapsed for this review because older adults frequently utilize all four park typologies interchangeably for daily recreational and therapeutic activities within their living environments.

Furthermore, the broader term “green space” was strategically included to capture relevant empirical studies that investigate identical spatial settings (i.e., residential neighborhood parks) but employ more generic nomenclature in their titles or abstracts. To prevent over-inclusion, any generic “green space” literature retrieved through this strategy was strictly screened against the exclusion criteria, ensuring that large-scale wilderness areas, agricultural lands, or private gardens were systematically excluded, and only publicly accessible urban green spaces within residential contexts were retained.

Search terms were organized around four concept groups:

“community park” OR “urban park” OR “public park” OR “neighborhood park” OR “green space*.”

“therapeutic landscape” OR “therapeutic qualities” OR “therapeutic value” OR “healing landscape.”

“older adult” OR “older people” OR “senior*.”

preference* OR perception* OR attitude* OR experience* OR satisfaction.

### 3.2 Eligibility criteria

The eligibility criteria are summarized in Table 1. Studies were included if they met all of the following criteria:

### 3.3 Selection process

**Figure 1:**
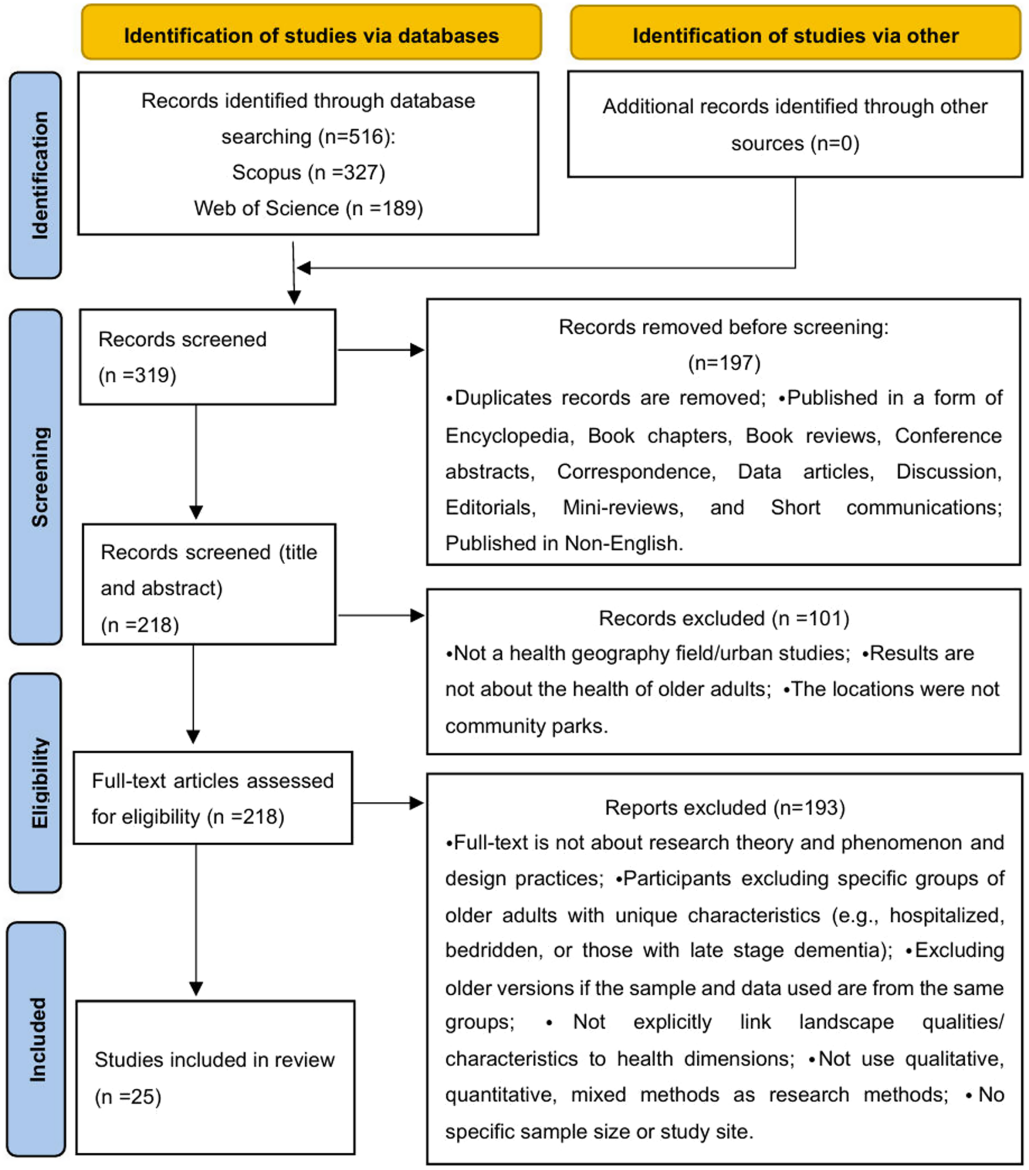
PRISMA flow diagram.

The literature search was conducted using database specific field tags applied to titles, abstracts and keywords. Automated filters at the platform level restricted the initial pool to English language journal articles published from database inception to 31 July 2025 (Figure 1).

The initial search returned 516 records. Before screening, 197 records were removed, comprising duplicates, non-article publications (encyclopedia entries, book chapters, book reviews, conference abstracts, correspondence, data articles, discussions, editorials, mini reviews, and short communications), and non-English publications. A total of 319 records were retained for the screening phase.

Title and abstract screening were independently conducted by two reviewers against the predefined eligibility criteria, with disagreements resolved through discussion until consensus was reached. Records were excluded if they fell outside the fields of health geography and urban studies, if their outcomes did not address the health of older adults, or if the study sites were not community parks. This stage yielded 101 exclusions, leaving 218 reports for full text assessment.

Full text screening was then performed against the inclusion and exclusion criteria specified in Figure 1. A total of 193 reports were excluded for the following reasons: full text not addressing research theory, phenomena, or design practice; studies focusing on clinical or institutionalized older adult subgroups (e.g., hospitalized patients, bedridden individuals, or those with late stage dementia) rather than community dwelling older adults; redundant publications drawing on the same sample and dataset as earlier versions; absence of an explicit link between landscape qualities or characteristics and health dimensions; designs other than qualitative, quantitative, or mixed methods; and unspecified sample size or study site. Twenty five studies satisfied all inclusion criteria and were retained for synthesis. The full search was completed on 31 July 2025, and the selection process is summarized in Figure 1.

### 3.4 Data extraction

A data extraction form (Supplementary material S2), was used to collect data on author(s), publication year, country/region, methods, sample size, main findings. Two reviewers extracted data independently, with discrepancies resolved through discussion and a third reviewer consulted where consensus could not be reached.

### 3.5 Study risk of bias assessment

Methodological quality was assessed independently by two reviewers using the Mixed Methods Appraisal Tool (MMAT) (29), which accommodates qualitative, quantitative, and mixed-methods designs. Following the MMAT user guide, studies meeting all five design specific criteria were classified as high quality (HQ), those meeting three or four as medium quality (MQ), and those meeting two or fewer as poor quality (PQ). Disagreements were resolved through discussion, with a third reviewer consulted where necessary. Across the 25 included studies, 20 (80%) were rated as high quality and 5 (20%) as medium quality, none fell into the poor quality category. The distribution of study designs comprised 12 mixed methods studies, 6 qualitative studies, 5 quantitative non randomized studies, and 2 quantitative descriptive studies. As part of our strict quality threshold and retention criteria, the assessment was used to audit study validity rather than mechanically exclude relevant literature. Since all retrieved papers achieved at least a medium quality rating, all 25 studies were retained for the final narrative synthesis to maximize data richness. Full quality ratings are provided in Supplementary material S3.

## 4 Results

### 4.1 Study selection

Figure 1 shows the PRISMA flow diagram of the selection of studies. Following the review of titles and abstracts, a total of 101 articles were excluded, leaving 218 articles for full-text review. Based on the eligibility criteria, the focus was on reviewing sample selection, research methods, outcome presentation, and ultimately, only 25 articles met the inclusion criteria.

### 4.2 General characteristics of the included studies

This section presents the general characteristics of the 25 included studies. The synthesis proceeds along three dimensions: the geographical and temporal distribution of the evidence base, the methodological approaches employed, and the sociodemographic heterogeneity of the older adult populations examined. The first two dimensions describe the external features of the research field, whereas the third addresses the internal variation that shapes how therapeutic landscape effects are reported across subgroups.

#### 4.2.1 Geographical and temporal distribution

**Figure 2:**
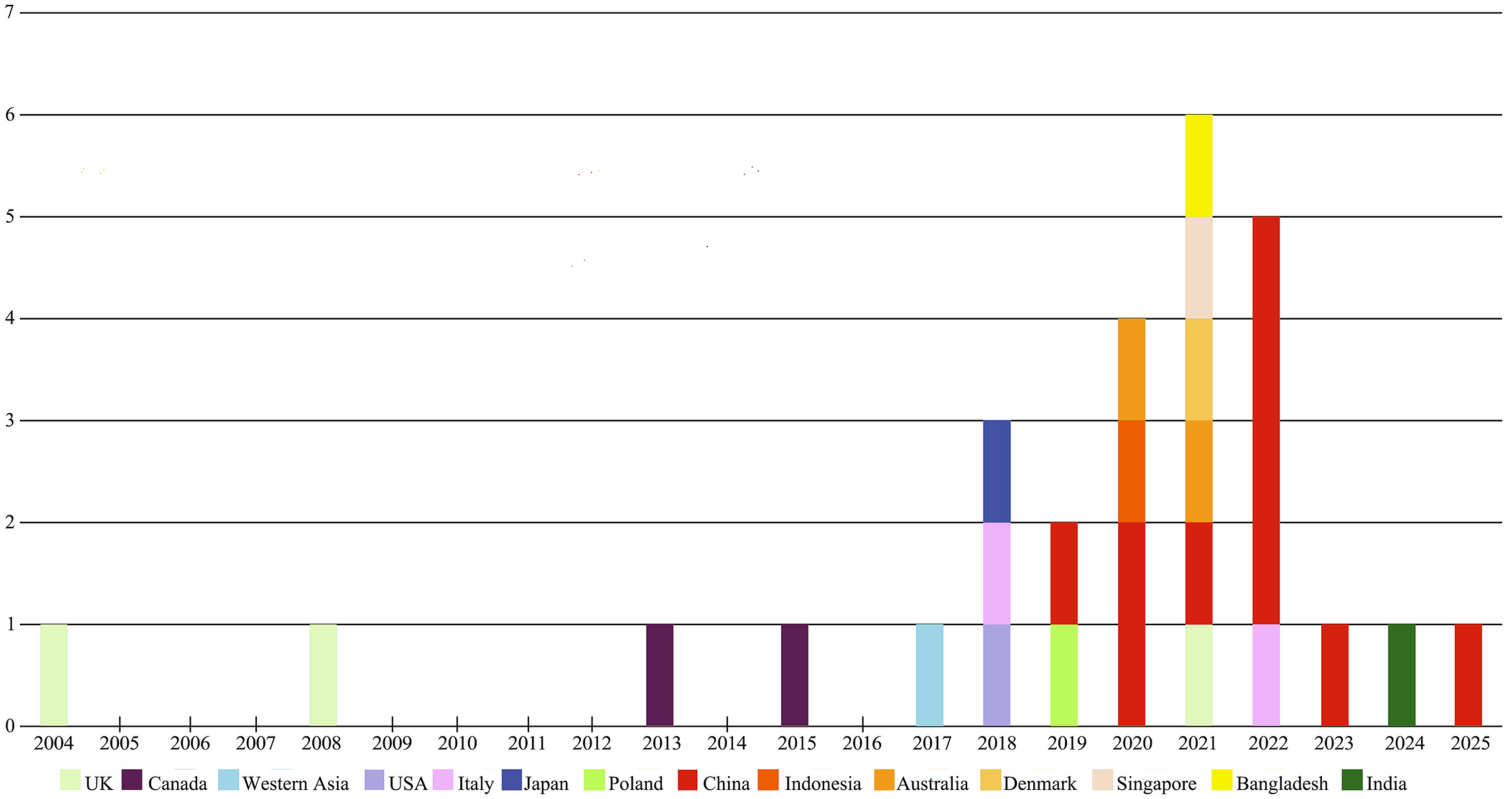
Geographical scope based on systematic review and year of publication.

The 25 included studies were published between 2004 and 2025, originating from 14 countries across four continents (Figure 2). The temporal pattern reflects two phases that correspond to shifts in research orientation. The early phase (2004–2017) consisted of sparse studies in the United Kingdom and Canada (22, 24, 30, 31, 50), which established the qualitative foundations of the therapeutic landscape framework, often through ethnographic work in marginalized communities. The later phase (2018–2025) saw a marked acceleration in volume and a geographical shift toward Asia, with China contributing 10 of the 25 studies and reaching peak output in 2022 (*n* = 4).

Three research orientations can be distinguished beneath this surface pattern. Studies from China (*n* = 10) were conducted in medium-to-large cities such as Shanghai, Guangzhou, Nanjing, Jinan, Chengdu, Hangzhou, and Shenzhen, and concentrated on the relationships between specific landscape features and psychological or emotional outcomes (32–36, 49, 52). Studies from South and Southeast Asia (37, 53) addressed more foundational concerns of accessibility, basic infrastructure, and barrier-free design, reflecting contexts where the baseline provision of age-appropriate public space remains contested. Studies from Western and Northern Europe, North America, and Australia (22, 24, 31, 38, 47, 50, 51, 54) typically engaged with socioeconomically stratified or disadvantaged neighborhoods, examining how park environments interact with social isolation, neighborhood deprivation, and life-course transitions.

The included studies also varied in residential setting, spanning dense inner-city districts (52), aged suburban housing estates (39), rural resettlement communities (40), and cross-city comparisons stratified by socioeconomic status (41). This variation indicates that therapeutic landscape research has progressed from documenting such effects in selected Western contexts toward examining how those effects vary across cultural, economic, and spatial settings, a progression with direct implications for the transferability of findings to the Chinese community park context.

#### 4.2.2 Methodological characteristics

**Figure 3:**
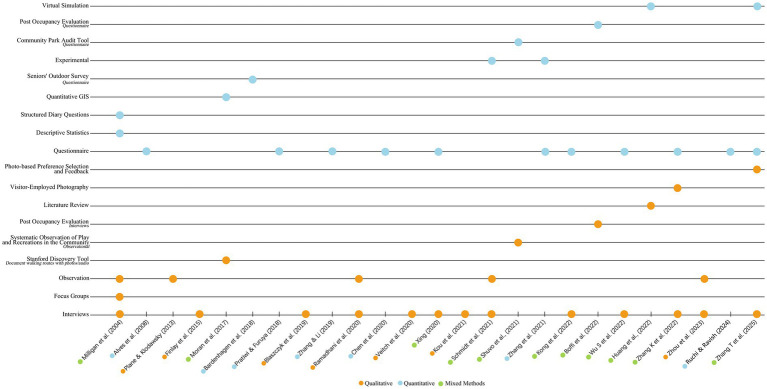
Method summary based on systematic review.

The 25 studies adopted mixed methods (*n* = 11), qualitative (*n* = 7), and quantitative (*n* = 7) designs (Figure 3), and the choice of method aligned closely with the research orientations identified above. Qualitative work, mainly from the United Kingdom, Canada, Australia, and Indonesia (22, 24, 42, 43, 50, 53, 54), relied on in-depth interviews, walking interviews, and participant observation to capture lived experience and the symbolic dimensions of place. Quantitative work, dominated by Chinese and Japanese studies (32, 34, 35, 39), was largely questionnaire-based and concerned with statistical associations between landscape attributes and self-reported outcomes; the Indian study (37) followed the same template, surveying 198 older adults to test associations between green space, walking paths, social spaces, and three domains of well-being.

Mixed methods designs predominated from 2021 onwards and introduced methodological innovations not seen in earlier work, including virtual reality simulation (47, 48), community park audit tools (41), behavior mapping with SOPARC (38, 40), visitor-employed photography (49), and Isovist analysis with image-based emotional rating (52). The combination of interviews and questionnaires remained the most common pairing [*n* = 5; (33, 40, 44, 49, 52). This methodological diversity expands the evidentiary base but creates two practical limitations for synthesis: outcome measures and landscape attribute classifications differ substantially across studies, and the technologically intensive Chinese studies and the experience-grounded Western studies generate findings that are not always directly comparable.

#### 4.2.3 Sociodemographic variations

**Figure 4:**
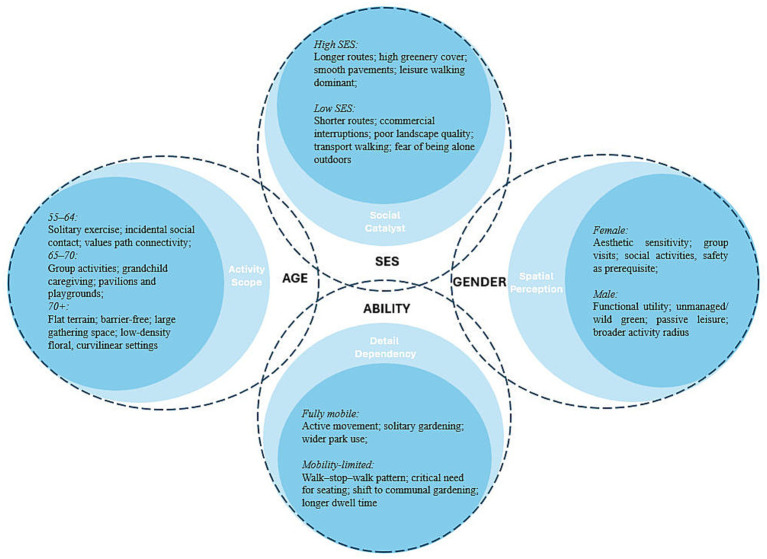
Sociodemographic variations in therapeutic landscape preferences among older adults.

Older adults are not a homogeneous population. The reviewed literature indicates that older adult subgroups with different sociodemographic characteristics exhibit distinct preferences for therapeutic landscape features (Figure 4). Four sociodemographic dimensions emerged from the included studies: age, gender, physical and functional ability and socioeconomic status.

##### 4.2.3.1 Age differences

Ramadhani et al. (53) subdivides older adults into the young-old (55–64), the middle-old (65–70), and the oldest-old (70 and above). The young-old typically exercise alone or in the early morning and prefer incidental social interaction. The middle-old, whose functional capacity has begun to decline and who frequently take on grandchild-caring responsibilities, tend toward group activities with companions. The oldest-old, despite being the most mobility-constrained, are the most regular park users and place the most demanding requirements on the physical environment, including level terrain, barrier-free facilities, and large spaces for collective activities. Zhang et al. (52) found that emotional responses to green space characteristics vary across age groups. Older adults aged 60 and above tend to experience negative emotions in overly dense or geometrically uniform green spaces, preferring quieter environments with a higher proportion of flowering plants, lower vegetation density and natural curves. Zhang et al. (49) compared the perceived cultural ecosystem services (CESs) of younger and older adults in detail, finding that older adults place greater value on bequest value, therapeutic value, social relations, and cultural heritage, including plazas, pavilions, and playgrounds, primarily for watching and caring for children.

##### 4.2.3.2 Gender differences

Zhang et al. (52) found that women demonstrate higher emotional sensitivity to natural environments. Women are more likely to express positive emotions in well-connected and well-maintained green spaces, placing greater value on esthetic qualities and the social dimension of landscapes. Men, by contrast, tend toward neutral emotional responses, prioritizing the functional utility of green spaces, such as commuting shortcuts, or preferring undeveloped, wilder forest environments. Wu et al. (40), in a study of resettlement communities in China, found that older women tend to participate in socially interactive and domestically oriented physical activities, including chatting, grandchild care, square dancing, and household chores, while older men prefer leisure activities such as card games, chess, watching, and gardening. Overall, women show greater dependence on outdoor spaces within the immediate community, whereas men maintain a wider daily activity radius. Women generally place greater importance on safety and are more likely to visit parks accompanied by family or friends rather than alone. In Western contexts and among certain vulnerable groups, fear of sexual harassment at night constitutes a primary barrier to female park use. Kong et al. (33), however, identified a culturally specific pattern in China, where well-established community policing means that as many as 68% of middle-aged and older women are willing to visit parks at night in groups for square dancing and similar activities.

##### 4.2.3.3 Physical and functional ability

Alves et al. (30) examined how functional ability shapes older adults preferences for park attributes, finding significant differences between mobility-limited and mobile older adults. Those with mobility difficulties demonstrate particularly high dependence on seating, requiring not only adequate rest facilities within the park but also seats along the route en route to the park to support a walk-stop-walk travel pattern. Milligan et al. (24) found that older adults who previously preferred solitary gardening actively transition to communal gardening projects as physical capacity declines. Blaszczyk et al. (42) found that older adults with limited physical function, such as those using walking aids or wheelchairs, require longer travel times to reach parks, and once they arrive, tend to stay longer given the high cost of the journey, making shelter from sunshine and rain a more pressing requirement. Wu et al. (40) and Zhou et al. (36) confirm that as physical capacity becomes more restricted, older adults’ activity patterns shift toward socially interactive behaviors such as chatting and passive leisure activities such as watching.

##### 4.2.3.4 The impact of socioeconomic status

Shuvo et al. (41) highlights significant inequality in the distribution of green space quality driven by socioeconomic conditions and policy contexts. In Sydney, socioeconomically disadvantaged areas tend to have lower-quality parks, while Singapore demonstrates that strong public policy intervention can ensure high-quality parks even in disadvantaged neighborhoods. Overall, however, socioeconomic status (SES) remains the core determinant of access to green space resources. Moran et al. (31) compared the walking routes of older residents in high- and low-SES communities, finding that those in high-SES neighborhoods walk longer routes with higher green coverage and fewer commercial interruptions, with well-paved sidewalks more frequently cited as a walking facilitator. Low-income older residents, by contrast, face more commercial destinations and poorer landscape quality along their routes, a difference that translates into more leisure walking among affluent users and more transport-oriented walking among low-income users. In disadvantaged communities, older adults face a higher risk of social exclusion. Schmidt et al. (38), in a study of South Harbor, one of Copenhagen’s most deprived neighborhoods, found that physical improvements alone are insufficient for these residents, whose most powerful deterrent to park use is the fear of going out and finding no one there.

### 4.3 Synthesis of therapeutic landscape dimensions

**Figure 5:**
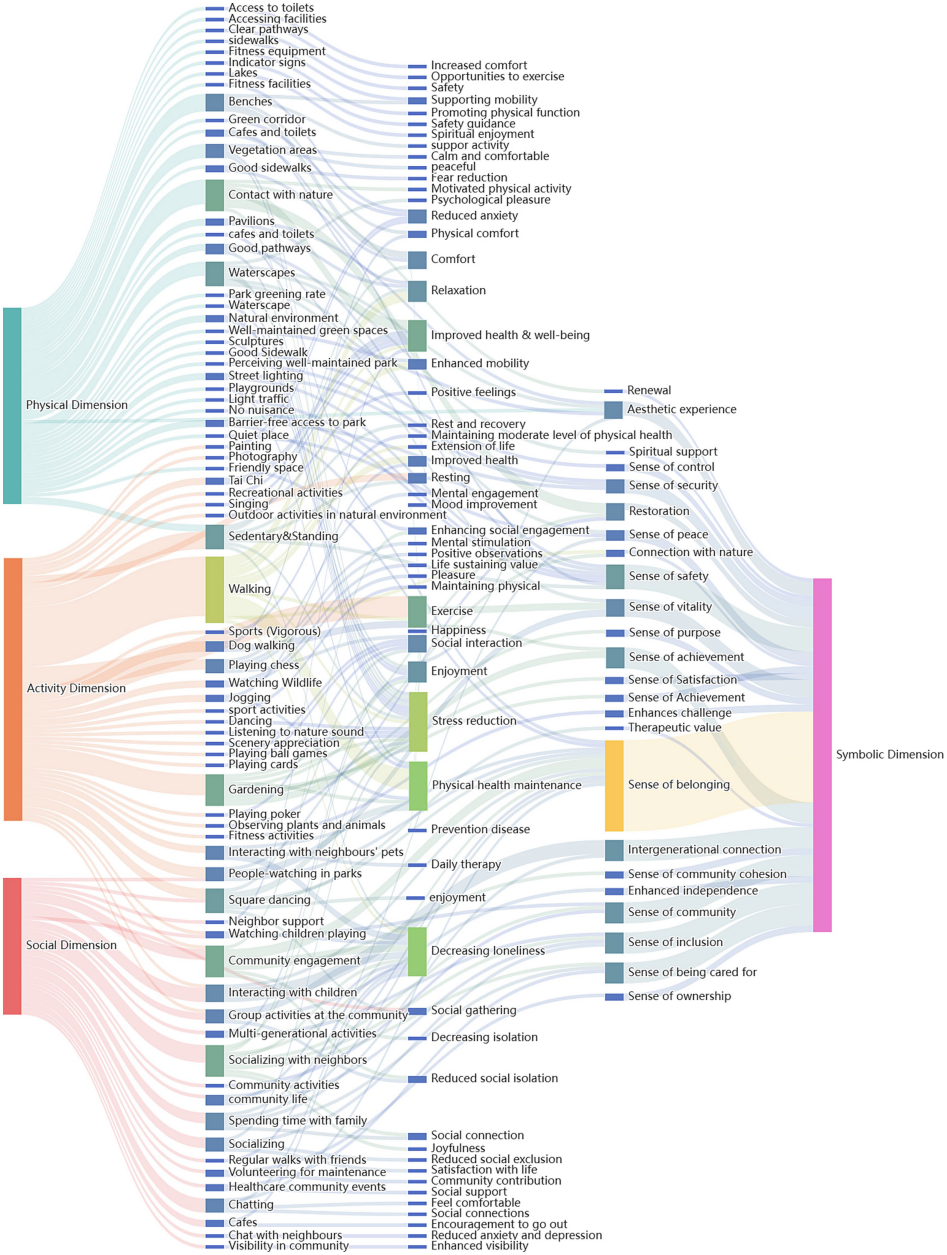
The relationship between therapeutic dimensions.

The complex relationships between environmental dimensions of community parks, older users’ activities, resultant health outcomes and symbolic dimensions of therapeutic experience were illustrated on Sankey diagram (Figure 5). The diagram synthesizes findings across the included studies, revealing four primary pathways: Physical Dimension, Activity Dimension, Social Dimension, through which specific elements and activities generated particular psychological perceptions, and these perceptions converged toward the Symbolic Dimension representing deeper therapeutic meanings.

#### 4.3.1 The physical dimension

The physical dimension encompasses environmental features-built environment characteristics and natural elements. Contact with nature emerged as a fundamental element (22, 24, 34, 37, 43, 48, 42), connecting to improved health and well-being, restoration (related to symbolic dimension), and stress reduction. Natural elements including waterscapes (22, 31, 44, 42) and vegetation areas (32, 35, 36, 52, 53) were frequently mentioned by older participants. Waterscapes demonstrated therapeutic effects related to the symbolic dimension, particularly in fostering a sense of peace, sense of vitality, spiritual support and esthetic experience. Vegetation areas generated psychological perceptions, notably stress reduction. Infrastructure features support functional therapeutic outcomes. Benches (22, 35, 39, 44, 51) and seating areas facilitate rest and recovery while enabling social interaction. Accessibility features include clear pathways (53), barrier-free access (37, 50, 53) and good sidewalks (22, 42) connect to enhanced mobility, safety, and increased comfort. Basic amenities such as toilets and cafes (30, 43, 54) and fitness equipment (35, 40) provide opportunities to exercise and promote physical function. Well-maintained environments, including park greening rate, well-maintained green spaces and good pathways, demonstrate connections to esthetic experience (50), sense of safety (32), indicating that environmental quality influences both functional and psychological outcomes. However, deficiencies in these physical features can interrupt the therapeutic process. Damaged, slippery, or uneven surfaces along the route from home to the park generate fear of falling that may lead older adults to abandon outdoor activity altogether in favor of indoor exercise (22, 31). The continuity of pedestrian routes is further disrupted by illegally parked vehicles and overgrown vegetation, forcing slow-moving older adults onto motorized lanes and increasing traffic safety risks (22, 31, 42). At the micro-spatial level, steep terrain without barrier-free facilities combined with insufficient seating along routes prevents those with declining physical capacity from compensating through a walk-stop-walk pattern, directly shrinking their activity radius (38, 43). Landscape deterioration, including over-hardened plazas, unmaintained vegetation, dry waterscapes, and sensory pollution such as litter, communicates abandonment and signals neglect by urban management (31, 33).

#### 4.3.2 The activity dimension

The activity dimension reveals the behavioral repertoire of older park users. Walking constitutes the predominant therapeutic activity, evidenced across multiple studies (22, 24, 30–33, 36, 38–40, 43, 44, 48, 42, 53–54), connecting to relaxation, stress reduction, physical health maintenance. The substantial representation of walking in the Sankey diagram reflects its accessibility and multifunctional therapeutic value and as a foundational therapeutic activity in community park settings. Gardening activities demonstrated strong associations with the symbolic dimension. Older participants perceived it as conducive to achieving a sense of achievement, sense of satisfaction, sense of purpose and restoration (22, 24, 40, 51, 52). Sedentary and standing behaviors connect to rest and recovery (30, 32, 38, 43, 47, 51), indicating that passive park use holds therapeutic significance. More vigorous activities including exercise and sports activities flow toward happiness, physical health maintenance, and sense of vitality (related to symbolic dimension). Nature-engaged activities such as watching wildlife (30, 49), listening to nature sounds (36) and scenery appreciation connect to pleasure, mental engagement, and sense of peace (related to symbolic dimension) highlight the restorative potential of direct nature contact. Functional conflicts between user groups can, however, transform these therapeutic activities into sources of tension. Fear of collisions with cyclists on shared paths, competition for limited fitness equipment, and interference between square dancing groups and chess players have been reported as significant psychological stressors that interrupt the therapeutic value of park activities (33, 36, 43).

#### 4.3.3 The social dimension

Social engagement operates through diverse pathways. The diagram reveals a clear pattern: the social dimension demonstrates strong connections to the symbolic dimension. Socializing with neighbors (33, 34, 39, 43, 50), intergenerational interactions (33, 39, 51, 53, 54), and community engagement (31, 50, 51) emerged as the most frequent avenues for promoting social life among older participants. Particularly, interacting with children and observing children at play were associated with decreased loneliness and enhanced sense of belonging and sense of vitality. Socializing with neighbors and community engagement activities demonstrated pathways to social gathering, thereby enabling older participants to experience deeper spiritual perceptions manifested as sense of belonging, sense of community cohesion, sense of being cared for, sense of ownership, and sense of inclusion. The complexity of these pathways reveals that social engagement in parks operates through multiple mechanisms to enhance social well-being. Yet social environment barriers can be more destructive than physical deficiencies. Even where physical facilities are adequate, fear of nuisance and crime, including vandalism, signs of drug use, and the presence of marginal groups, directly determines whether older adults are willing to use parks at all, particularly causing women and older adult users to leave during low-light hours or specific time periods (30, 31, 36, 50). For older residents in disadvantaged communities, the deepest barrier is fear of social isolation, where the prospect of going out and finding no one there, without social support systems or collective activities, becomes a form of psychological confinement that prevents the formation of social well-being (38).

#### 4.3.4 The symbolic dimension

The right side of the diagram demonstrates convergence toward the symbolic dimension, representing deeper existential and psychological meanings. From the perspective of older adults, the symbolic dimension encompasses sense of belonging, intergenerational connection, sense of community cohesion, enhanced independence, sense of community, sense of inclusion, sense of being cared for, and sense of ownership. These symbolic meanings represent the highest-order therapeutic outcomes, transcending immediate physical or social benefits to encompass existential dimensions of place attachment, identity, and community integration.

## 5 Discussion

### 5.1 Therapeutic landscapes and older adult health as a cyclical co-production process

**Figure 6:**
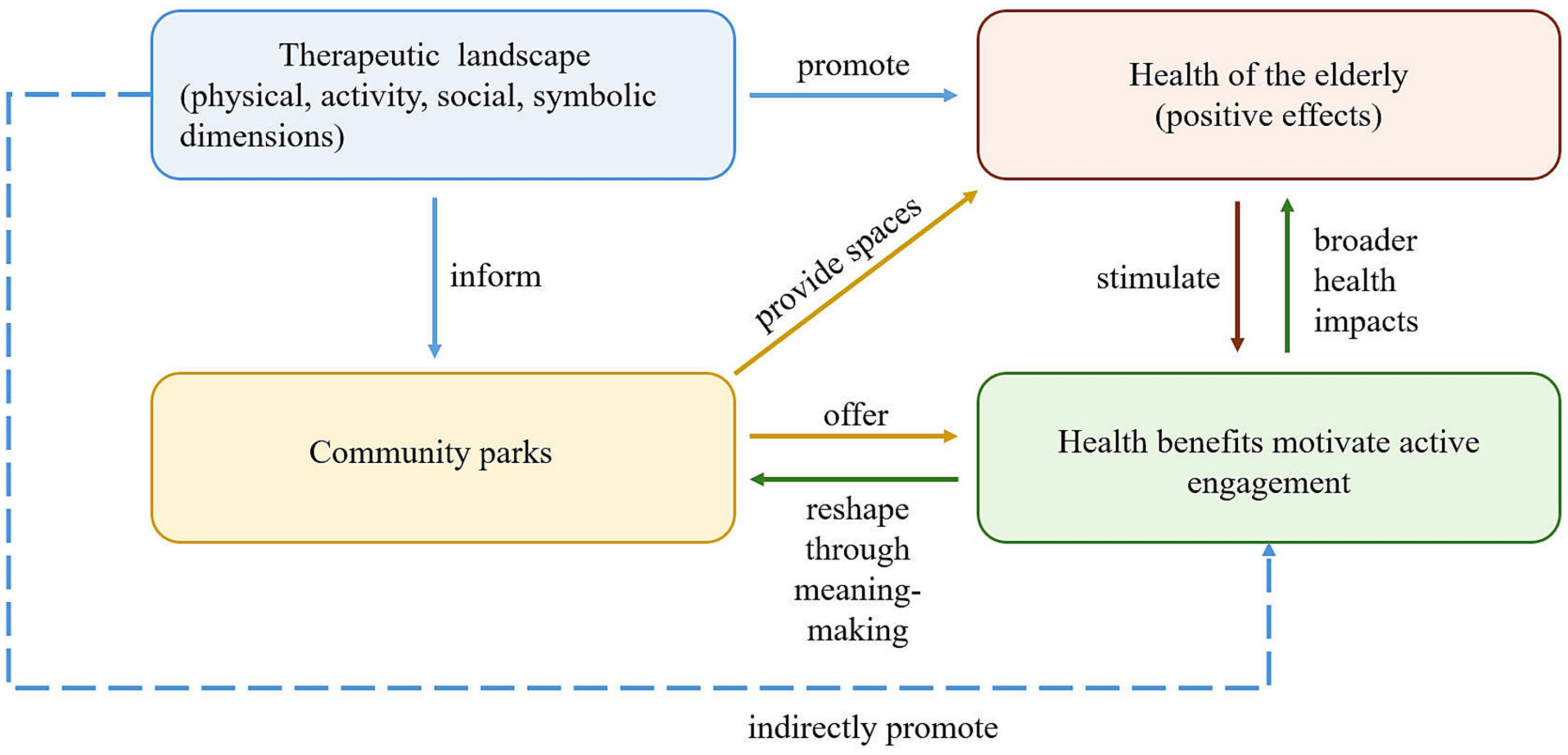
Cyclical co-production of therapeutic landscapes and older adults’ health in community parks.

Existing studies on therapeutic landscapes and older adults’ health have largely examined this relationship as unidirectional, where environmental characteristics shape health outcomes and the analysis stops there. The evidence synthesized from the 25 reviewed articles, however, points to a more dynamic process. As illustrated in Figure 6, the relationship between therapeutic landscape and older adults’ health operates as a self-reinforcing cycle rather than a linear sequence of cause and effect.

The cycle begins with therapeutic landscape across physical, activity, social and symbolic dimensions informing how community parks function as health-enabling environments. These parks offer affordances that older adults engage with, producing measurable health benefits. What the reviewed literature collectively suggests, though, is that this engagement does not terminate at the point of health gain. Health benefits motivate older adults to return, participate more frequently, and invest socially and emotionally in the space like a pattern consistent with place attachment theory (45) and the concept of environmental agency and belonging (46) Through sustained engagement, older adults reshape the park itself via meaning-making: appropriating spaces for collective practice, embedding cultural routines and transforming functionally ordinary park elements into symbolically charged therapeutic resources. This reshaped environment then produces broader health impacts, completing the cycle and reinforcing its next iteration.

This cyclical co-production process recognizes that older adults are not passive recipients of therapeutic affordances but active agents in the shaping of therapeutic space. The cyclical process (Figure 6) also implies that disruptions at any point, whether through deterioration of physical infrastructure, loss of social programming, or displacement of culturally embedded practices, can interrupt the entire process with compounding consequences for older adults’ health.

### 5.2 The hierarchical priority framework of therapeutic landscapes

**Figure 7:**
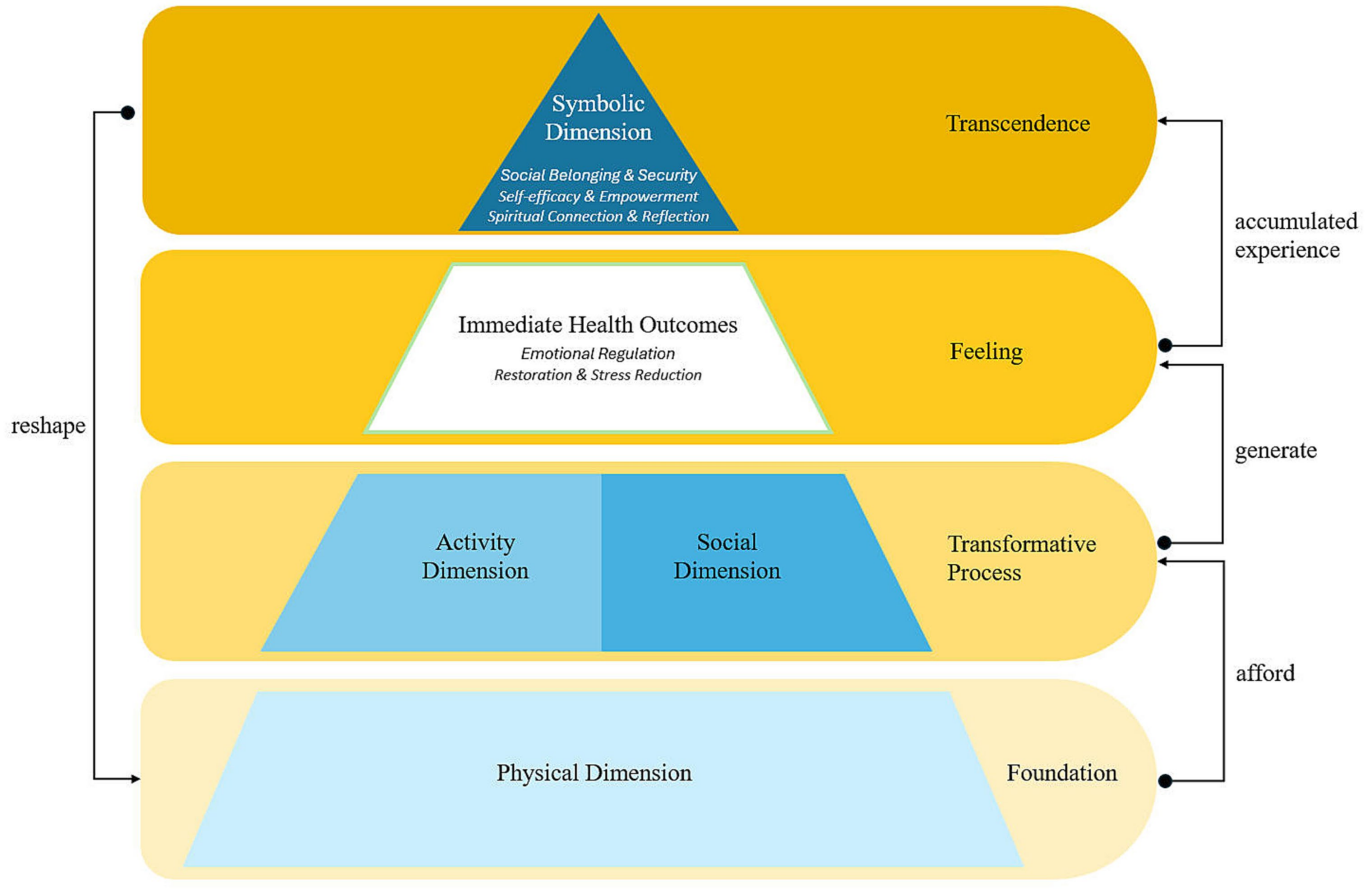
The hierarchical priority framework of therapeutic landscapes.

Gesler's (2) foundational articulation of therapeutic landscapes identified physical, social, and symbolic dimensions as the core dimensions through which places acquire healing properties. Subsequent scholarship has extended this framework in various directions, yet the three dimensions have largely been treated as parallel constituents of therapeutic experience rather than as elements operating in a structured sequence. Including existing theoretical frameworks in environmental gerontology, Wahl et al.'s (46) integrative model and Lawton and Nahemow’s (55) ecological model of aging, have advanced understanding of how environments shape health outcomes in later life. The hierarchical priority framework proposed here, as illustrated in Figure 7, represents a departure from that tradition. Drawing on the synthesis of evidence across the 25 reviewed studies, the framework argues that these dimensions are not equivalent in their therapeutic function: they operate through a hierarchy in which each layer enables and conditions the next and in which the symbolic dimension occupies a qualitatively distinct apex that cannot be reached without the layers beneath it.

As illustrated in Figure 7, the framework comprises four hierarchically ordered layers. Physical dimension constitutes the foundation, functioning not as a direct source of therapeutic benefit but as the necessary precondition for it. Accessible pathways, seating, shade structures and green space afford the conditions under which activity and absence of nuisance become possible (30, 51). This distinction between infrastructure as endpoint and infrastructure as enabler is consequential: the reviewed evidence consistently shows that physical amenities alone do not produce durable health outcomes unless they successfully support sustained behavioral and social engagement. Once these functional and safety prerequisites are established, the landscape can effectively support the activity and social dimension. Within these secure and accessible physical settings, older adults engage in diverse performative enactments, ranging from stationary collective exercises to passive leisure observation, thereby fostering reciprocal social interactions and mutual support (36). Simultaneously older adults park users consistently reported that deficiencies in physical features, whether poor pathway maintenance, insufficient seating, or inadequate shelter, constrained their ability to engage in the activities through which health benefits are actually generated (22, 38, 43). The physical environment is therefore necessary but not sufficient.

The activity and social dimensions together constitute the transformative process in the second layer. It is through walking, gardening, group exercise, spontaneous social encounters, and intergenerational interaction that the affordances of the physical environment are converted into health-relevant experience (24, 51). These two dimensions are represented as a single layer in the framework because the reviewed evidence points consistently to their interdependence. Activities generate social opportunities, and social contexts in turn shape the nature and intensity of activity engagement (36, 40). This layer is where the park begins to function therapeutically in a meaningful sense, and it is where the greatest diversity of individual preference and sociodemographic variation is observed.

The third layer, immediate health outcomes, captures the feelings generated through sustained activity and social engagement. These include emotional regulation, restoration and stress reduction (35, 48). This layer occupies a structurally distinct position in the framework in that it serves an intermediary rather than a terminal function. This layer is not a component drawn from existing therapeutic landscape theory but is instead proposed and foregrounded in this framework as a conceptually significant contribution. The health outcomes it captures are real and meaningful in their own right; however, their fuller significance lies in what they set in motion. Through repeated visits, accumulated affective and restorative experience at this level gradually converges toward the symbolic dimension above.

The symbolic dimension at the apex of the framework represents transcendence beyond individual health outcomes through long periods of repetition and deep experience. Sense of belonging, self-efficacy and empowerment, spiritual connection and reflection, and social belonging and security are not simply more intense versions of the feelings in the layer below. They are qualitatively different in that they involve the collective transformation of a physical space into a place of shared meaning, identity, and community (31, 34, 50). This distinction is particularly consequential in Asian cultural contexts. The reviewed studies conducted in urban China consistently showed that the symbolic dimension was not a residual outcome of park use but its primary motivation for many older participants. The park was valued because it was the site of collective morning exercise, intergenerational care, and community ritual, practices through which older adults maintained their sense of purpose and social integration (33, 36, 40, 44). Gesler's (2) original framework acknowledged the symbolic environment as one of three dimensions but did not theorize its relationship to the other dimensions or its particular salience for aging populations embedded in specific cultural contexts. The framework proposed here addresses that gap by positioning the symbolic dimension not as one of three parallel components but as the apex of a hierarchy that is constituted through the accumulation of experience across all layers beneath it.

The left-side arrow in Figure 7, labeled reshape, connects the symbolic layer back to the physical dimension and links this framework to the cyclical co-production process presented in section 4.1. As older adults develop place attachment and a sense of ownership through accumulated symbolic experience, they actively reshape the physical and social conditions of the park through informal appropriation, collective programming and spatial meaning-making (45, 46). The therapeutic landscape is therefore not a static property of the environment, but an ongoing achievement of its users and the hierarchy is not a one-way progression, but a dynamic structure sustained by feedback between its layers.

Gesler’s (2) original conceptualization of therapeutic landscapes defined them as places with an enduring reputation for healing, a definition that implicitly assumes therapeutic properties to be stable and inherent. The evidence synthesized in this review challenges that assumption fundamentally. What the barriers documented across the reviewed studies reveal is that the therapeutic quality of a community park is not a fixed attribute but a fragile condition, one that can be undermined at any dimensions of the hierarchy and whose maintenance requires as much deliberate effort as its creation.

The overwhelming majority of studies in this field, including most of the 25 reviewed here, are designed to identify what makes environments therapeutic, not what makes them fail. This positive orientation has produced a rich understanding of affordances and health-enabling conditions, but it has also generated a blind spot: the assumption that removing barriers is simply the inverse of adding benefits. The reviewed evidence suggests otherwise. Physical deterioration does not merely reduce therapeutic potential; it actively produces a countersignal. Poorly maintained vegetation, dry waterscapes, and visually neglected plazas communicate abandonment, eroding the sense of place identity that is the foundation of symbolic experience (31, 33). For older users who have invested years of accumulated experience in a park, physical neglect is not a neutral absence of quality but a rupture in the place attachment that constitutes the symbolic layer of therapeutic experience. Gesler's (2) framework has no adequate account of this condition, because it was built on the study of places that already possessed an established therapeutic identity. The community parks documented in this review are ordinary everyday spaces whose therapeutic potential is contingent, not guaranteed, and whose symbolic dimension depends entirely on the sustained presence of an active user community.

### 5.3 Sociodemographic variations in therapeutic landscape experience

The therapeutic potential of a community park is not an intrinsic property of its design but is differentially realized depending on who is using it and under what conditions.

The oldest-old are simultaneously the most physically constrained and the most regular park users, with the most demanding requirements for physical infrastructure (53). This suggests that for this group, park attendance has become a necessary daily ritual rather than discretionary leisure. In Wahl et al.’s (46) terms, the park has become the primary site through which belonging is maintained when the home environment can no longer sustain it. Infrastructure failures therefore represent not mere inconveniences but disruptions to the only remaining mechanism through which the oldest-old sustain their connection to the symbolic dimension.

The gender dimension is insufficiently captured by safety perception alone. Wu et al. (40) and Kong et al. (33) together reveal that women’s spatial boundedness through domestic responsibility and caregiving makes the community park their primary, and sometimes only, site of social engagement outside the home. This means the social and symbolic dimension carry greater therapeutic effect for older women than for men. Kong et al.'s (33) finding that older women in China actively used parks at night for square dancing, enabled by community policing infrastructure, illustrates how culturally specific governance conditions can unlock therapeutic access that Western research assumptions would predict to be foreclosed, underscoring the importance of contextualizing therapeutic landscape theory rather than applying it universally.

Physical functional decline intensifies rather than diminishes the need for therapeutic landscape access, while simultaneously raising the threshold required to achieve it. Milligan et al.'s (24) observation that older adults transition from solitary to communal gardening as individual capacity declines suggests that the social dimension becomes more therapeutically critical precisely when physical capacity is most compromised. If the social and symbolic dimension of therapeutic experience becomes more important as functional ability declines, then design interventions must ensure that physically constrained users are not confined to the lowest dimension of the hierarchy. A park that is physically accessible but socially empty offers little therapeutic value for functionally limited older users. For this group, an active social community is as essential as barrier-free infrastructure.

Shuvo et al. (41) and Moran et al. (31) together demonstrate that the quality of available park environments is itself stratified by neighborhood socioeconomic status. SES does not simply alter older adults’ aspirations for high-quality environments; it alters their baseline reality and the mechanisms through which therapeutic experience is achievable. For higher-SES older users, the park functions as an extension of esthetic and leisure life. For low-income older users, not only are personal resources more limited, but the parks available to them are less likely to possess the physical and social conditions necessary to support progression through the therapeutic hierarchy. For this group, the park becomes the only free refuge against loneliness, making the fear of social isolation, of arriving to find no one there, a more powerful deterrent than any physical deficiency (38).

This finding reframes the relationship between social programming and therapeutic landscape design. If the fear of being alone is a more powerful deterrent than poor physical conditions, then the social layer of the framework is not simply a product of good design; it is a precondition for any design to function therapeutically at all. Design interventions targeting low-SES communities must therefore move beyond improving physical infrastructure and treat social software, including community organizers, free programming, and social caretakers, as core components of therapeutic landscape design. The implication for Gesler's (2) original framework is significant: the symbolic dimension that Gesler identified as constitutive of therapeutic places cannot emerge in conditions of social fragmentation, regardless of the quality of the physical environment.

### 5.4 Implications for park design and policy

**Figure 8:**
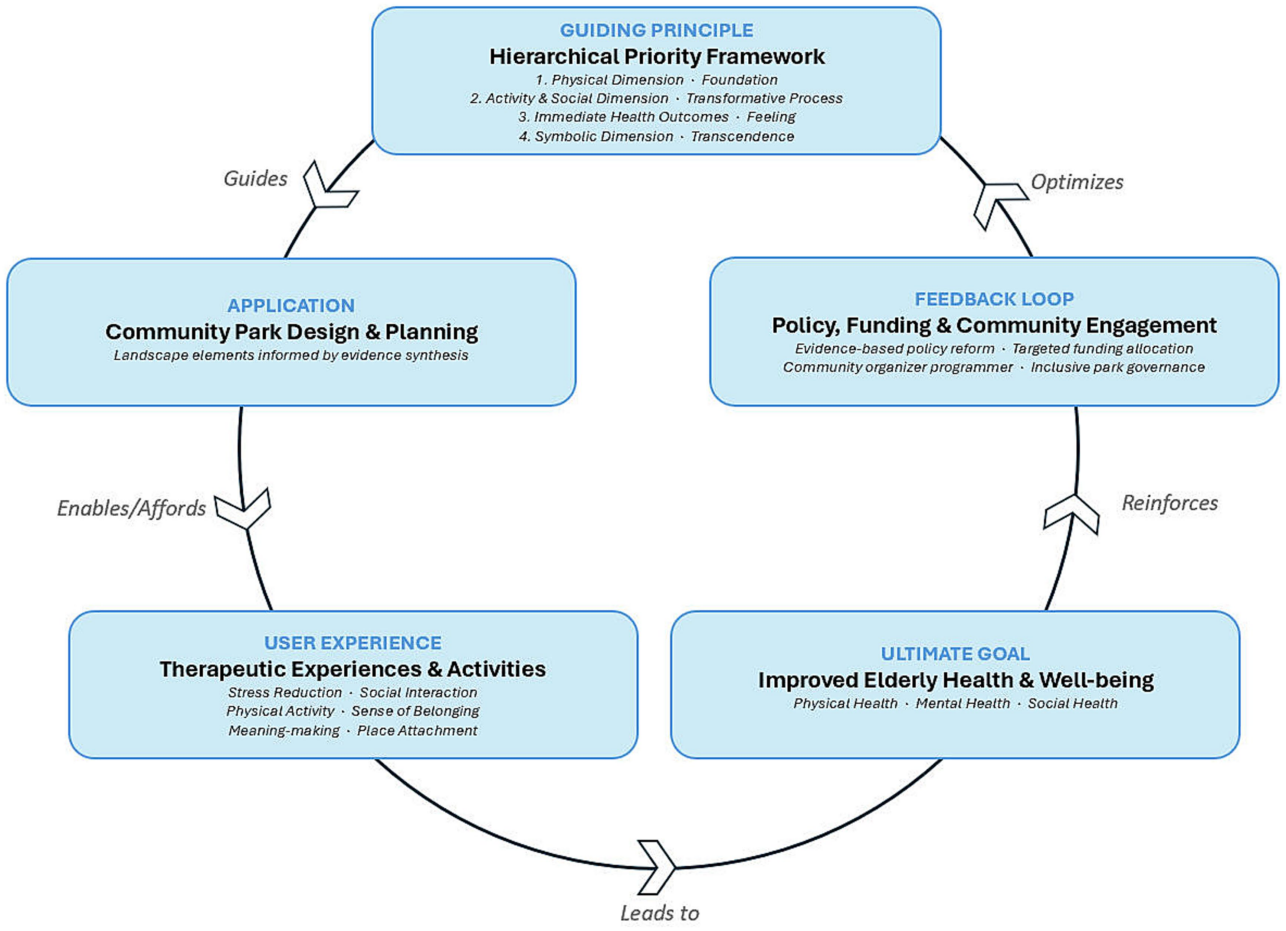
Implications of the hierarchical priority framework for age-friendly community park design and policy.

Translating theoretical findings into actionable urban interventions requires a systemic approach. As illustrated in Figure 8, the Hierarchical Priority Framework serves as a guiding principle for community park design, initiating a continuous operational cycle. Rather than adopting isolated design interventions, planners must recognize how physical improvements directly enable therapeutic activities, ultimately improving older adults’ health outcomes. Sustaining these benefits depends substantially on a feedback loop constituted by policy support, recurrent funding, and community engagement, ensuring that design practice remains responsive to the dynamic sociodemographic needs identified across this review. Therefore, designing a therapeutic landscape is not a problem that can be solved once, it is a condition that must be continuously maintained.

### 5.5 Limitations and future works

Several limitations of this review warrant acknowledgement. Although this review utilized Web of Science and Scopus as its comprehensive primary search databases, the exclusion of other specialized databases and the English-only screening criterion may introduce potential publication bias, potentially limiting the geographical representativeness of the synthesized evidence. The older population is treated with insufficient granularity across the included studies. Female participants outnumber male participants in most samples, and the majority of participants fall within the 60–69 age group, leaving the oldest-old and male older users underrepresented. Given the sociodemographic variations documented in Section 3.5, this sampling bias means the evidence base reflects a subset of the older population rather than its full range. Additionally, most included studies rely on self-reported perceptions of therapeutic experience, which may not fully capture the cumulative and symbolic dimensions of park use that emerge only through long-term engagement.

Future research should address these gaps in three directions. First, broader database coverage and inclusion of non-English publications, particularly in Chinese, Japanese, and Korean, would help reduce selection and language bias. Second, studies that deliberately stratify samples by age sub-group, gender, functional ability, and socioeconomic status would allow more precise identification of which landscape features produce therapeutic benefits for which populations. Third, longitudinal designs are needed to test the cyclical relationship, particularly whether accumulated experience produces symbolic outcomes over time and how disruptions to the cycle affect older adults’ health. Last, the Hierarchical Priority Framework proposed here requires empirical validation across diverse cultural and urban contexts to assess its generalizability beyond the settings represented in the current evidence base.

## 6 Conclusion

This systematic review addressed how therapeutic landscape dimensions collectively operate in community parks to support older adults’ health. Synthesizing evidence from 25 studies reveals that therapeutic experiences are actively co-produced rather than passively received. The proposed Hierarchical Priority Framework demonstrates that physical, social, immediate health outcomes, and symbolic dimensions function through a sequence of dependency. Within this sequence, the symbolic dimension represents the highest level of therapeutic experience, achievable only through accumulated engagement.

Theoretically, the findings challenge frameworks derived predominantly from Western contexts. The evidence, heavily concentrated in urban China, shows that the symbolic dimension is a primary motivation for park use among many older populations, rather than a peripheral outcome. This highlights a critical need to adapt therapeutic landscape theory to Asian communities. Practically, these insights offer direct guidance for age-friendly design and public health practice. Because therapeutic value accumulates through sustained use, providing physical park infrastructure is insufficient. Public health policies and park management must continuously support social programming to maintain long-term health benefits.

While the findings should be interpreted considering the inherent limitations of the existing empirical base (such as uneven sample demographics and reliance on self-reported perceptions), this review provides a structured, foundational stepping stone. To advance this field, future research must empirically validate and cross-culturally adapt the Hierarchical Priority Framework across wider global contexts using longitudinal designs and stratified cohorts.

## Data Availability

The original contributions presented in the study are included in the article/Supplementary material, further inquiries can be directed to the corresponding author/s.
