# Supplementary material for: Developing a hierarchical priority framework for therapeutic landscapes in age-friendly community parks: a systematic review
Source: Front Public Health. 2026 Jul 1;14:1839266. doi: 10.3389/fpubh.2026.1839266 (PMC13368659; doi:10.3389/fpubh.2026.1839266)
Supplement: Supplementary file 1 [file Supplementary_file_1.DOCX]

Supplementary Material

# Literature Search Strategy

Databases: Web of Science Core Collection (all indexes: SCI-EXPANDED, SSCI, A&HCI, ESCI, CPCI-S, CPCI-SSH, BKCI-S, BKCI-SSH, ESCI) and Scopus databases for journal articles published from database inception up to 31 July, 2025.

| Step | Core Operations & Content | | | Implementation Protocol |
| --- | --- | --- | --- | --- |
| Search strategy | Database | Search Date | Complete Search String | Automated filtering |
|  | Web of Science | 31 July 2025 | TS=("community park" OR "urban park" OR "public park" OR "neighbourhood park" OR "green space*") AND TS=("therapeutic landscape" OR "therapeutic qualities" OR "therapeutic value" OR "healing landscape") AND TS=("older adult" OR "older people" OR "senior*") AND TS=(preference* OR perception* OR attitude* OR experience* OR satisfaction) | Language: English Document Type: Article Timespan: Inception to 31 July, 2025 |
|  | Scopus | 31 July 2025 | TITLE-ABS-KEY(("community park" OR "urban park" OR "public park" OR "neighbourhood park" OR "green space*") AND ("therapeutic landscape" OR "therapeutic qualities" OR "therapeutic value" OR "healing landscape") AND ("older adult" OR "older people" OR "senior*") AND (preference* OR perception* OR attitude* OR experience* OR satisfaction)) | Language: English Document Type: Article Timespan: Inception to 31 July, 2025 |
| Title and abstract screening | Duplicate records across the two databases were removed. Two reviewers independently screened the titles and abstracts of the remaining records against the eligibility criteria, blinded to authors and affiliations. Records outside human geography, health geography, landscape architecture, and urban research were excluded. | | | WOS and Scopus built-in filters: language = English; document type = journal article |
| Full-text screening | Two reviewers independently assessed full texts against inclusion and exclusion criteria. Reviewed sample selection, methods, and outcome reporting. Disagreements resolved through discussion. | | | Blind screening conducted manually by two independent reviewers based on Eligibility criteria (Table 1) |
| Selection reporting | Documented the flow of records from identification to inclusion, including reasons for exclusion at each stage. | | | PRISMA flow diagram |
| Data extraction | Two reviewers independently extracted data, with discrepancies resolved through discussion and a third reviewer consulted when consensus could not be reached. | | | Standardized extraction form covering: author(s), publication year, country, study aims, sample characteristics, data collection methods, main findings, limitations |
| Quality Assessment | Evaluated the methodological quality and risk of bias of all studies across qualitative, quantitative, and mixed-methods designs. Two reviewers independently appraised methodological quality. Disagreements resolved through discussion, a third reviewer consulted when needed. | | | Mixed Methods Appraisal Tool, MMAT (Supplementary Material 2) |

# Data Extraction Form

| Study ID | Author(s) & Publication year | Country / Region | Methods | Samples size (older adults) | Main findings |
| --- | --- | --- | --- | --- | --- |
| S01 | Milligan et al. (2004) | Northern England, UK | Mixed methods: Focus groups, semi-structured interviews, observational,  structured diary questions  Descriptive Statistics | Male 13  Female 6 | Demonstrated that participating in communal gardening acts as a long-term emotional sanctuary for older individuals, mitigating senile loneliness across physical, psychological, and social dimensions of the therapeutic landscape. |
| S02 | Alves et al. (2008) | England, Scotland, Wales, UK | Quantitative: Questionnaire | Male 114  Female 84 | Indicated that the inclusion of public restrooms yields the highest marginal utility for elderly park visitors, a requirement that supersedes the demand for cafes. Additionally, dense arboreal greenery contributes to a noticeable elevation in positive affect. |
| S03 | Plane & Klodawsky (2013) | Ottawa, Ontario, Canada, city center | Qualitative: Observation | Male 0  Female9 | Employed participant observation to delineate how the social attributes and physical features of public green spaces uniquely foster a sense of community belonging and psychological well-being, focusing particularly on female cohorts. |
| S04 | Finlay et al. (2015) | Metro Vancouver, Canada | Qualitative: Interviews | 19 | Examined the immediate and cumulative impacts of blue-and-green spaces on geriatric health. While an isolated walk provides immediate emotional relief, a sustained, long-term walking routine is necessary to generate cumulative benefits that systematically alleviate chronic health conditions. |
| S05 | Moran et al. (2017) | Haifa, Israel | Mixed methods: Quantitative GIS, Stanford Discovery Tool | Male 14  Female45 | Revealed that perceived walking barriers, such as uneven pavements, a lack of benches, and high social deprivation, significantly compress the daily activity space of older adults. |
| S06 | Bardenhagen et al. (2018) | Milan, Italy & Texas, USA | Quantitative: Seniors' Outdoor Survey (SOS Tool). | 116 | Cross-nationally validated the Systemic Outdoor Environment Evaluation Tool (SOS Tool) for older adults, confirming that senior citizens prioritize park accessibility, convenience, safety, and barrier-free designs. Their preferences also extend to whether these environments possess therapeutic or healing properties capable of accommodating health-promoting activities and social interaction. |
| S07 | Pratiwi & Furuya (2018) | Tokiwadaira, Matsudo, Japan | Quantitative: Questionnaire | Male121  Female99 | Observed that individuals over the age of 70 constitute more than 57% of visitors in Japanese community parks, suggesting that spatial convenience and barrier-free microclimate design are vital to sustaining socialization among the oldest old. |
| S08 | Blaszczyk et al. (2019) | Warsaw, Poland | Qualitative: Interviews | Male7  Female8 | Conducted qualitative in-depth interviews with elderly users of urban parks in Warsaw, revealing that ergonomic backrests, shaded canopies, and visual or mobility aids directly dictate the dwell time of wheelchair users and visually impaired seniors. |
| S09 | Zhang & Li (2019) | Nanjing, China | Quantitative: Questionnaire. | 192 | Constructed a mediation model linking neighborhood environments to quality of life. The empirical results showed that well-paved sidewalks, barrier-free infrastructure, and a sense of social security within community green spaces indirectly enhance overall subjective well-being by facilitating daily physical activity. |
| S10 | Ramadhani et al. (2020) | Surabaya City, Indonesia | Qualitative: Observation, Interviews | 98 | Investigated elderly park requirements and concluded that older demographics exhibit a pronounced demand for spatial adaptations tailored to sensory decline, highlighting an urgent need for multi-sensory rehabilitative landscapes and gently sloped, barrier-free pathways. |
| S11 | Chen et al. (2020) | Guangzhou, China | Quantitative: Questionnaires | Male111  Female123 | Reported that high-quality outdoor green spaces and spatial openness within residential communities lessen negative affect among senior residents, fostering long-term benefits such as tranquility, relaxation, and rejuvenation. |
| S12 | Veitch et al. (2020) | Melbourne, Australia | Qualitative: Interviews | 30 | Utilized interviews to map out the "perfect park" from the perspective of older adults, characterized by abundant green buffers, secure walking paths, clean public restrooms, and zones completely segregated from vehicular traffic. |
| S13 | Xing (2020) | Taiyuan, China | Mixed methods: Questionnaire, Interviews | 147 | Found that older adults and youth are the primary users of community parks, utilizing these spaces predominantly for physical fitness, socialization, and leisure. Comfort, safety, and aesthetics emerge as pivotal elements in community park design, with users paying close attention to infrastructure, water features, and vegetation, while noting that environmental quality, seating facilities, and lighting systems require further optimization. |
| S14 | Kou et al. (2021) | Belfast, UK | Qualitative: Interviews | Male14  Female6 | Established that natural soundscapes, such as birdsong and flowing water, bolster psychiatric restoration in older adults, whereas anxieties induced by deteriorating hardscapes and graffiti trigger negative emotions. |
| S15 | Schmidt et al. (2021) | Copenhagen, Denmark | Mixed methods: Observations, Interviews | Male22  Female37 | Demonstrated that age-friendly micro-interventions in community green spaces, such as introducing additional seating and barrier-free pathways, lead to a substantial increase in both the daily outdoor visitation frequency and the duration of continuous exercise among senior citizens. |
| S16 | Shuvo et al., (2021) | Sydney, Australia/ Singapore/Dhaka, Bangladesh | Mixed methods: Systematic Observation of Play and Recreations in the Community (SOPARC), Community Park Audit Too (CPAT) | 71 | Identified a strong positive correlation between high-quality parks equipped with fitness installations and accessible restrooms, and the engagement of older adults in vigorous daily physical activities. |
| S17 | Zhang et al. (2021) | Shanghai, China | Quantitative: Questionnaires, Experiments | Male260  Female280 | Identified a strong positive correlation between high-quality parks equipped with fitness installations and accessible restrooms, and the engagement of older adults in vigorous daily physical activities. |
| S18 | Kong et al. (2022) | Jinan City, China | Mixed methods: Questionnaire, Interviews | 542 | Discovered that upgrading hardscapes with age-friendly, barrier-free alternatives, such as replacing rough brick pavements with highly elastic rubber tracks, significantly mitigates fall anxiety and eases overall heart rate stress among elderly users. |
| S19 | Boffi et al. (2022) | Milan, Italy | Mixed methods: Post-Occupancy Evaluation (POE), Interviews, Questionnaire | 71 | Determined that when older adults reside within specific multi-sensory landscape Points of Interest (POVs) in community gardens—such as under wisteria pergolas or shaded seating—their psychological restoration and pleasure scores are significantly higher than in non-natural, hardscaped environments. |
| S20 | Wu S et al. (2022) | Ningbo, China. | Mixed methods: Questionnaires, Interviews | Male40  Female43 | Found that elderly physical exercise is predominantly governed by spatial convenience and site security. Notably, female seniors prefer central plazas for group dancing (60.6%), an activity characterized by collective social attributes. |
| S21 | Huang et al., (2022) | Hainan, China | Mixed methods: Virtual Simulation, Literature review | Male15  Female15 | Discovered that when older adults view seating arrangements equipped with backrests sheltered under tree canopies, their psychological stress dissipates rapidly, as evidenced by the sharpest declines in electromyography (EMG) and galvanic skin response (GSR) metrics. |
| S22 | Zhang K et al. (2022) | Chengdu, China | Mixed methods: Visitor-Employed Photography (VEP), Questionnaires, Interviews | Male60  Female60 | Indicated that older adults exhibit a distinctly higher cultural-spiritual attachment and perceived cognitive clustering toward traditional Chinese pavilions, long corridors, pergolas, and bridges. |
| S23 | Zhou et al. (2023) | Beijing and Huainan, China | Qualitative: Interviews, Observation | Male18  Female24 | Highlighted that proactive "elderly exercise leaders" actively transform otherwise neglected urban dead-zones into highly inclusive therapeutic spaces by organizing localized, static activities such as square dancing and choral singing. |
| S24 | Ruchi & Kumar (2024) | Noida, India | Quantitative: Questionnaire | Male114  Female84 | Established a highly significant causal link between immersion in natural parks and feeling refreshed and rejuvenated; frequent daily visits (a 39.39% daily attendance rate) markedly improve scores on the WHO-5 Well-Being Index. |
| S25 | Zhang T et al. (2025) | Hong Kong | Mixed method: Virtual simulation, Questionnaires Interviews, Photo-based preference selection/feedback analysis | Male24  Female0 | Revealed that within high-density, high-rise urban contexts, parks featuring high vegetation enclosure, distinct naturalness, and robust accessibility substantially delay cognitive decline and alleviate daily anxiety among the elderly. |

# Methodological Quality Assessment

| Study | Study Design | S1 | S2 | 5.1 | 5.2 | 5.3 | 5.4 | 5.5 | Quality |
| --- | --- | --- | --- | --- | --- | --- | --- | --- | --- |
| Milligan et al. (2004) | Mixed methods | Y | Y | Y | Y | Y | Y | Y | HQ |
|  |  | **S1** | **S2** | **3.1** | **3.2** | **3.3** | **3.4** | **3.5** | **Quality** |
| Alves et al. (2008) | Quantitative non-randomized | Y | Y | Y | Y | Y | Y | Y | HQ |
|  |  | **S1** | **S2** | **1.1** | **1.2** | **1.3** | **1.4** | **1.5** | **Quality** |
| Plane & Klodawsky (2013) | Qualitative | Y | Y | Y | Y | Y | Y | Y | HQ |
|  |  | **S1** | **S2** | **1.1** | **1.2** | **1.3** | **1.4** | **1.5** | **Quality** |
| Finlay et al. (2015) | Qualitative | Y | Y | Y | Y | Y | Y | Y | HQ |
|  |  | **S1** | **S2** | **5.1** | **5.2** | **5.3** | **5.4** | **5.5** | **Quality** |
| Moran et al. (2017) | Mixed methods | Y | Y | Y | Y | Y | CT | Y | HQ |
|  |  | **S1** | **S2** | **4.1** | **4.2** | **4.3** | **4.4** | **4.5** | **Quality** |
| Bardenhagen et al. (2018) | Quantitative descriptive | Y | Y | Y | CT | Y | Y | Y | HQ |
|  |  | **S1** | **S2** | **3.1** | **3.2** | **3.3** | **3.4** | **3.5** | **Quality** |
| Pratiwi & Furuya (2018) | Quantitative non-randomized | Y | Y | CT | Y | Y | N | Y | MQ |
|  |  | **S1** | **S2** | **1.1** | **1.2** | **1.3** | **1.4** | **1.5** | **Quality** |
| Blaszczyk et al. (2019) | Qualitative | Y | Y | Y | Y | Y | Y | Y | HQ |
|  |  | **S1** | **S2** | **4.1** | **4.2** | **4.3** | **4.4** | **4.5** | **Quality** |
| Zhang & Li (2019) | Quantitative descriptive | Y | Y | Y | CT | Y | Y | Y | HQ |
|  |  | **S1** | **S2** | **1.1** | **1.2** | **1.3** | **1.4** | **1.5** | **Quality** |
| Ramadhani et al. (2020) | Qualitative | Y | Y | Y | Y | Y | Y | Y | HQ |
|  |  | **S1** | **S2** | **3.1** | **3.2** | **3.3** | **3.4** | **3.5** | **Quality** |
| Chen et al. (2020) | Quantitative non-randomized | Y | Y | CT | Y | Y | N | Y | MQ |
|  |  | **S1** | **S2** | **1.1** | **1.2** | **1.3** | **1.4** | **1.5** | **Quality** |
| Veitch et al. (2020) | Qualitative | Y | Y | Y | Y | Y | Y | Y | HQ |
|  |  | **S1** | **S2** | **5.1** | **5.2** | **5.3** | **5.4** | **5.5** | **Quality** |
| Xing (2020) | Mixed methods | Y | Y | Y | Y | N | CT | N | MQ |
|  |  | **S1** | **S2** | **1.1** | **1.2** | **1.3** | **1.4** | **1.5** | **Quality** |
| Kou et al. (2021) | Qualitative | Y | Y | Y | Y | Y | Y | Y | HQ |
|  |  | **S1** | **S2** | **5.1** | **5.2** | **5.3** | **5.4** | **5.5** | **Quality** |
| Schmidt et al. (2020) | Mixed methods | Y | Y | Y | Y | Y | Y | Y | HQ |
|  |  | **S1** | **S2** | **5.1** | **5.2** | **5.3** | **5.4** | **5.5** | **Quality** |
| Shuvo et al., (2021) | Mixed methods | Y | Y | Y | Y | Y | Y | Y | HQ |
|  |  | **S1** | **S2** | **3.1** | **3.2** | **3.3** | **3.4** | **3.5** | **Quality** |
| Zhang et al. (2021) | Quantitative non-randomized | Y | Y | Y | N | Y | Y | Y | HQ |
|  |  | **S1** | **S2** | **5.1** | **5.2** | **5.3** | **5.4** | **5.5** | **Quality** |
| Kong et al. (2022) | Mixed methods | Y | Y | Y | Y | Y | Y | Y | HQ |
|  |  | **S1** | **S2** | **5.1** | **5.2** | **5.3** | **5.4** | **5.5** | **Quality** |
| Boffi et al. (2022) | Mixed methods | Y | Y | Y | Y | Y | Y | Y | HQ |
|  |  | **S1** | **S2** | **5.1** | **5.2** | **5.3** | **5.4** | **5.5** | **Quality** |
| Wu S et al. (2022) | Mixed methods | Y | Y | Y | Y | Y | Y | Y | HQ |
|  |  | **S1** | **S2** | **5.1** | **5.2** | **5.3** | **5.4** | **5.5** | **Quality** |
| Huang et al., (2022) | Mixed methods | Y | Y | Y | Y | Y | CT | N | MQ |
|  |  | **S1** | **S2** | **5.1** | **5.2** | **5.3** | **5.4** | **5.5** | **Quality** |
| Zhang K et al. (2022) | Mixed methods | Y | Y | Y | Y | Y | Y | Y | HQ |
|  |  | **S1** | **S2** | **1.1** | **1.2** | **1.3** | **1.4** | **1.5** | **Quality** |
| Zhou et al. (2023) | Qualitative | Y | Y | Y | Y | Y | Y | Y | HQ |
|  |  | **S1** | **S2** | **3.1** | **3.2** | **3.3** | **3.4** | **3.5** | **Quality** |
| Ruchi, & Kumar (2024) | Quantitative non-randomized | Y | Y | CT | Y | CT | Y | Y | MQ |
|  |  | **S1** | **S2** | **5.1** | **5.2** | **5.3** | **5.4** | **5.5** | **Quality** |
| Zhang T et al. (2025) | Mixed methods | Y | Y | Y | Y | Y | Y | Y | HQ |

Appraisal Key: Y=Yes; N=No; CT=Can't tell.

HQ=High Quality; MQ=Medium Quality; PQ=Poor Quality.
